# High-Resolution Optical Measurement of Cardiac Restitution, Contraction, and Fibrillation Dynamics in Beating vs. Blebbistatin-Uncoupled Isolated Rabbit Hearts

**DOI:** 10.3389/fphys.2020.00464

**Published:** 2020-05-26

**Authors:** Vineesh Kappadan, Saba Telele, Ilija Uzelac, Flavio Fenton, Ulrich Parlitz, Stefan Luther, Jan Christoph

**Affiliations:** ^1^Research Group Biomedical Physics, Max Planck Institute for Dynamics and Self-Organization, Göttingen, Germany; ^2^German Center for Cardiovascular Research (DZHK e.V.), Partnersite Göttingen, Göttingen, Germany; ^3^School of Physics, Georgia Institute of Technology, Atlanta, GA, United States; ^4^Institute for the Dynamics of Complex Systems, University of Göttingen, Göttingen, Germany; ^5^Department of Pharmacology, University Medical Center Göttingen, Göttingen, Germany; ^6^Department of Cardiology and Pneumology, University Medical Center Göttingen, Göttingen, Germany

**Keywords:** fluorescence imaging, optical mapping, motion tracking, motion correction, computer vision, ventricular fibrillation, electromechanics, Blebbistatin

## Abstract

Optical mapping is a high-resolution fluorescence imaging technique, that uses voltage- or calcium-sensitive dyes to visualize electrical excitation waves on the heart surface. However, optical mapping is very susceptible to the motion of cardiac tissue, which results in so-called *motion artifacts* in the fluorescence signal. To avoid motion artifacts, contractions of the heart muscle are typically suppressed using pharmacological excitation-contraction uncoupling agents, such as Blebbistatin. The use of pharmacological agents, however, may influence cardiac electrophysiology. Recently, it has been shown that numerical motion tracking can significantly reduce motion-related artifacts in optical mapping, enabling the simultaneous optical measurement of cardiac electrophysiology and mechanics. Here, we combine ratiometric optical mapping with numerical motion tracking to further enhance the robustness and accuracy of these measurements. We evaluate the method's performance by imaging and comparing cardiac restitution and ventricular fibrillation (VF) dynamics in contracting, non-working vs. Blebbistatin-arrested Langendorff-perfused rabbit hearts (*N* = 10). We found action potential durations (APD) to be, on average, 25 ± 5% shorter in contracting hearts compared to hearts uncoupled with Blebbistatin. The relative shortening of the APD was found to be larger at higher frequencies. VF was found to be significantly accelerated in contracting hearts, i.e., 9 ± 2*Hz* with Blebbistatin and 15 ± 4*Hz* without Blebbistatin, and maintained a broader frequency spectrum. In contracting hearts, the average number of phase singularities was *N*_*PS*_ = 11 ± 4 compared to *N*_*PS*_ = 6 ± 3 with Blebbistatin during VF on the anterior ventricular surface. VF inducibility was reduced with Blebbistatin. We found the effect of Blebbistatin to be concentration-dependent and reversible by washout. Aside from the electrophysiological characterization, we also measured and analyzed cardiac motion. Our findings may have implications for the interpretation of optical mapping data, and highlight that physiological conditions, such as oxygenation and metabolic demand, must be carefully considered in *ex vivo* imaging experiments.

## 1. Introduction

Optical mapping is a high-resolution fluorescence imaging technique, which has been used in numerous studies to visualize electrical impulse phenomena, such as action potential waves, on the surface of intact isolated hearts. Due to its high spatial resolution, the technique has several advantages over contact-electrode measurements, including the ability to resolve APD dispersion and heterogeneity (Wu et al., 2002; Mironov et al., 2008) and visualize electrophysiological vortex waves during arrhythmias (Davidenko et al., 1992; Gray et al., 1998; Witkowski et al., 1998). Optical mapping has furthermore been used to measure cardiac electrical restitution at high spatial resolutions (Banville et al., 2004; Choi et al., 2004). However, until recently, one of the major drawbacks of optical mapping has been the necessity to uncouple cardiac excitation from contraction in order to avoid so-called motion artifacts. To avoid these artifacts, optical mapping studies have relied on pharmacological excitation-contraction uncoupling agents such as Blebbistatin (Dou et al., 2007; Fedorov et al., 2007; Farman et al., 2008). Blebbistatin inhibits actin-myosin interactions in cardiomyocytes, thereby decoupling electrophysiology from mechanics and suppressing the heart's contractile motion. Blebbistatin has been used extensively in the field because of its ease-of-use and efficacy in preventing motion artifacts; however, also in large part out of necessity, as the only alternative approach to reduce motion-artifacts involved mechanical restriction of the heart's motion. With the adaptation of computer vision techniques, with which motion in optical mapping videos can be tracked and compensated numerically, motion artifacts no longer pose a limitation in optical mapping and the imaging technique has been used to measure simultaneously cardiac electrophysiology and surface mechanics (Zhang et al., 2016; Christoph et al., 2017, 2018; Christoph and Luther, 2018). Moreover, optical mapping has recently been used in conjunction with high-speed 3D ultrasound to study electromechanical tissue dynamics during ventricular arrhythmias (Christoph et al., 2018). Nevertheless, despite the advances, further validation is required. For instance, it has yet to be determined whether the technique is sufficiently accurate to measure electrophysiological parameters such as action potential durations, and consequently cardiac electrical restitution on the strongly deforming heart surface.

Here, we combine optical mapping, using voltage-sensitive fluorescent dyes, with marker-free numerical motion tracking and ratiometric imaging. Firstly, we reduced motion artifacts to the greatest possible extent by tracking the motion of the heart wall and retrieving electrophysiological signals in a co-moving coordinate system. Secondly, we further reduced residual motion artifacts by using ratiometric imaging, exciting the voltage-sensitive dye Di-4-ANEPPS at two wavelengths, and retrieving and analyzing optical signals from two separate emission bandwidths, effectively compensating undesired effects caused by inhomogeneous illumination post-tracking. We used the imaging technique to measure cardiac APD restitution and fibrillation dynamics in Langendorff-perfused isolated rabbit hearts with and without Blebbistatin. Overall, we provide a comparison of contracting and non-contracting isolated hearts in a Langendorff-perfusion environment, and discuss the imaging technique's performance in measuring motion artifact-free optical maps and optical traces of cardiac electrophysiology.

## 2. Materials and Methods

### 2.1. Tissue Preparation

New Zealand White rabbits (*N* = 10, female, 6–10 months old, 2.5 − 3.5kg) were heparinized and anesthesized using 4.0ml Trapanal (single intraveneous injection, Thiopental-sodium solution, 50mg/kg) diluted in 10.0ml isotonic sodium chloride (NaCl). The hearts were excised rapidly and inserted into cardioplegic solution for temporary cessation of cardiac activity before being transferred into the experimental setup. All procedures regarding care and use of animals were carried out in accordance with German animal welfare laws and the recommendations of the Lower Saxony State Office for Customer Protection and Food Safety (LAVES) and the Federation of European Laboratory Animal Science Associations (FELASA). The protocol was approved by the Lower Saxony State Office for Customer Protection and Food Safety (LAVES).

### 2.2. Experimental Setup

**Figure 1:**
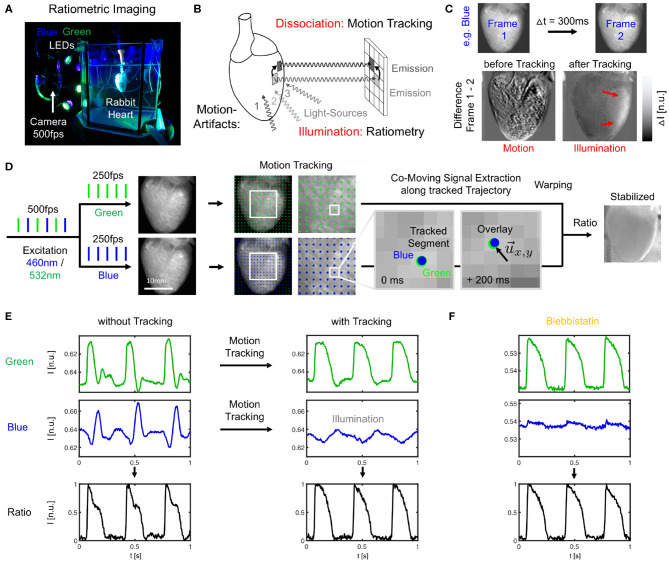
Dual-channel ratiometric optical mapping in combination with marker-free numerical motion tracking and motion-stabilization for imaging action potential waves on the contracting surface of isolated hearts. **(A)** *Ex-vivo* optical mapping setup with a single camera. **(B)** Sources for motion artifacts: dissociation-related and illumination-related motion artifacts are compensated with motion tracking and ratiometry, respectively. **(C)** Two types of motion artifacts visible when computing the difference between video frames. Before tracking, motion creates dissociation-related motion artifacts. After tracking, relative motion between heart surface and light sources (which now move in motion-stabilized video) causes residual intensity fluctuations or illumination-related motion artifacts (red arrows). **(D)** Imaging scheme with alternating blue (460 nm) and green (523 nm) illumination for excitation of Di-4-ANEPPS and exposure of consecutive video frames at 2 × 250 or 500 fps. Separate motion tracking in each channel (every *n*-th displacement vector shown, shifts are tracked in every pixel). Overlay of tissue coordinates tracked in blue and green channel, respectively, shows congruent motion through the video images, see also Supplementary Video 1. Combination of warped green and blue video images yields motion-stabilized ratiometric video image. **(E)** Optical traces (from 3 × 3 pixels) before and after tracking and/or ratiometry (pacing *CL* = 350 ms) in blue and green (inverted) channel, respectively. Ratiometry alone cannot completely remove all motion artifacts (bottom left), but only illumination-related motion artifacts. Numerical motion-stabilization alone cannot completely inhibit all motion artifacts (top right), but only dissociation-related motion artifacts. After tracking, ratiometric combination of green and blue signals yields an optical signal with very little residual motion artifacts. Due to the particular dye and filter properties, the relevant electrophysiological signal is in the green channel, whereas eventual intensity fluctuations in the blue channel (after tracking) are caused mostly by motion through an inhomogeneously illuminated scene. **(F)** Optical traces obtained with Blebbistatin in same heart. Ratiometry improves the signal with very little residual contractile motion, or after numerical motion-stabilization as in **(E)**.

Isolated rabbit hearts were positioned at the center of an eight-sided bath with 4 large (10 × 16 cm^2^) and 4 small (4 × 16 cm^2^) glass walls (see Figure 1A). The hearts were connected to a retrograde Langendorff-perfusion system with an aortic block and a bubble trap (Hugo-Sachs Apparatus, March-Hugstetten, Germany). Any fixation or mechanical pressure to the hearts was avoided to prevent compression of the coronary arteries. The hearts were only held in place by being connected with their aorta to the perfusion outflow. The bath was filled with ~ 2l of warm, oxygenated Tyrode solution (see Appendix for details), such that the hearts were completely immersed. In total, 15l of Tyrode solution was provided from a pre-heated reservoir, from where it was pumped into the heart and then constantly reperfused. The flow rate of the perfusion pump was set to 30 ml min^−1^. The perfusion pressure was set constantly to 50 ± 5mmHg and was regulated using a starling resistor and monitored throughout the experiment. The temperature of the Tyrode inside the bath was kept at a constant temperature of 37 ± 0.5°C (custom-made temperature control).

### 2.3. Blebbistatin

**Figure 2:**
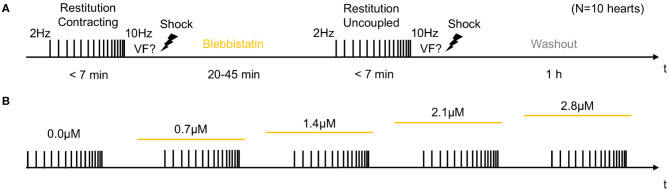
Experimental protocol for measurements of cardiac restitution using a dynamic pacing protocol in contracting and contraction-inhibited hearts uncoupled with Blebbistatin. **(A)** Measurement pre- and post-Blebbistatin in the same heart (total *N* = 10 hearts). Pacing from *f*_*p*_ = 2.0 to *f*_*p*_ = 10.0 Hz with progressively decreasing step size. After the first restitution measurement in the contracting heart, Blebbistatin was added at concentrations of up to 2.8μM. After 20–45 min, when Blebbistatin typically had suppressed all contractile motion, the second restitution measurement was performed in the contraction-inhibited heart. The reversal of the effect of Blebbistatin was verified in a washout experiment (after 1 h). **(B)** Subsequent increase of Blebbistatin concentration (0.0, 0.7, …, 2.8μM). The restitution measurement from **(A)** was performed for baseline and then repeated for all four concentrations in the same heart. The concentration was immediately increased after the previous measurement, and the next measurement was started after 30 min.

In the first half of the experiments, contracting hearts were imaged without administering Blebbistatin (see Figure 2A). In the second half of the experiments, Blebbistatin was administered to repeat the same measurements in uncoupled contraction-inhibited hearts. In all experiments, we used the Blebbistatin variant (−)/−Blebbistatin (Cayman Chemical Inc., USA). Prior to administering Blebbistatin, it was pre-diluted and stirred for at least 10 min in 300ml pre-heated 40°C warm Tyrode and then pumped directly into the aortic block's bubble trap (reaching 37°C) and from there into the heart. To avoid crystallization or the formation of precipitate that is associated with Blebbistatin (Swift et al., 2012), the Tyrode was filtered (5μm filter pore size) before being pumped into the heart. In some experiments (*N* = 2; see Figure 7), the concentration of Blebbistatin was step-wise increased from 0.7, 1.4, 2.1 to 2.8μM by adding in each step a bolus of 1ml of Blebbistatin directly to the 15l reservoir, see Figure 2B. After each increase of the concentration, we waited for about 30 min before starting the next measurement. In some experiments (*N* = 2), Blebbistatin was washed out toward the end of the experiment by perfusing hearts with Blebbistatin-free Tyrode for at least 60 min (see Figures 2A, 7D).

### 2.4. Ratiometric Optical Mapping

**Figure 3:**
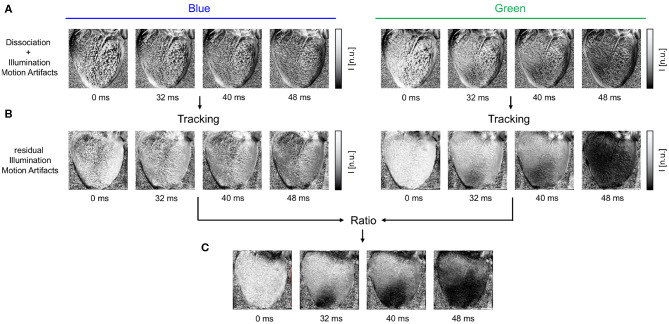
Motion artifacts and motion artifact reduction obtained through the combination of numerical motion tracking and dual channel excitation ratiometry using blue and green excitation light. **(A)** Optical maps showing mostly motion artifacts before tracking in uncompensated videos in blue and green channels, respectively. **(B)** Optical maps after motion tracking and stabilization. Dissociation-related motion artifacts are compensated and substantially reduced. The blue channel contains no electrophysiology-related signal, and the optical maps exhibit merely residual intensity modulations caused by relative motion between tissue and light sources, which persists after tracking (see also Figure 1C). The green channel exhibits an action potential wave propagating across the ventricular surface (dark: depolarized tissue). **(C)** Both dissociation- and illumination-related motion artifacts are compensated due to the combined, subsequent use of tracking and ratiometry. The optical maps show an essentially artifact-free action potential wave propagating across the deforming heart surface (dark: depolarized tissue). In all optical maps, the optical signal was normalized in each pixel over time (normalized units [n.u.], pixel-wise normalization) as the last processing step after numerical motion correction and ratiometry.

Optical mapping was performed using a single high-speed EMCCD camera (Evolve, 128 × 128 pixels, 16 bit dynamic range, Photometrics Inc., USA) and a wide-aperture lens (0.95/25 mm, Navitar, Japan) imaging field of views of about 1.5 × 1.5 cm^2^ at frame rates of 500Hz. The potentiometric dye Di-4-ANEPPS (500μl bolus injection into the bubble trap above the aortic block) was used for voltage-sensitive ratiometric imaging. Fluorescent emission light was filtered using a bandpass filter (590 ± 55nm, Omega Optical Inc., USA) mounted onto the lens in front of the camera. Ratiometric imaging (Knisley et al., 2000) was performed using two sets of high-power light-emitting diodes (LED; 3 blue LEDs: 460nm center wavelength, model LZ4-40B208; 3 green LEDs: 523nm center wavelength, model LZ4-40G108, both by LED Engin Inc., USA), and switching rapidly between the blue and green excitation light in every other frame (excitation ratiometry), similarly as previously described (Bachtel et al., 2011; Bourgeois et al., 2011). The synchronized, rapid switching was achieved using a custom-made electronic driver. The triggering of the two sets of LEDs was synchronized with the camera acquisition, such that odd and even video frames were illuminated with blue and green light, respectively (Figures 1A,D), resulting in a frame rate for each channel of *f*_*B*/*G*_ = 250Hz. The blue and green excitation light was further filtered by two sets of narrow bandpass filters (blue: 460 ± 5nm, Thorlabs, USA; green: 540 ± 12.5nm, Chroma Technology Corp., USA) and collimated with plano convex lenses (LA1951-A, Thorlabs, USA). To provide an even illumination of the heart surface, the light-emitting diodes were mounted (green, blue, green, …) on a ring-shaped post around the camera lens and were directed through one of the large glass walls of the aquarium at the tissue (see Figure 1A). All hearts were facing the camera with their anterior left ventricular surface. The camera's field of view covered the entire heart (see Figures 1, 3). The camera was controlled and optical mapping recordings were acquired using custom-made software. The videos were stored as two separate green and blue videos.

### 2.5. Motion Tracking and Motion-Stabilization

Two-dimensional in-plane motion was numerically tracked within the fluorescence video images with sub-pixel precision using optical flow-based algorithms, as previously described (Christoph et al., 2017; Christoph and Luther, 2018). The motion was tracked in the blue and green videos separately (see Figure 1D and Supplementary Video 1). Tissue displacements were computed in every pixel with respect to the first frame as the reference frame in each video *I*_*b*_(1) = *I*_*r, b*_ or *I*_*g*_(1) = *I*_*r, g*_, respectively. During pacing, the reference frame shows the tissue at the end of the diastolic interval shortly before the application of a pacing stimulus. During ventricular fibrillation, the reference frame shows the tissue in an arbitrary deformed state. Due to the illumination scheme, both reference frames *I*_*r, b*_ and *I*_*r, g*_ correspond to two consecutive frames with a temporal offset of 2ms in the interleaved video. Before tracking, the green and blue videos were normalized by their minimal and maximal values. Next, the local image contrast in the green and blue videos was enhanced, as described previously (Christoph and Luther, 2018). This pre-processing step ensures that tissue features can be reliably tracked throughout the image sequence, even as the action potential activity causes intensity in- or decreases. After tracking the contrast-enhanced green and blue videos, the original normalized blue and green videos were warped using the motion tracking data, as described previously (Christoph and Luther, 2018). The difference between pre- and post-tracking is illustrated in Figures 1C,E, 3A,B. Motion tracking was performed in the absence of markers attached to the heart surface and without user interaction, once a reference frame had been selected. The motion-stabilized green and blue videos were then combined by dividing one video by the other [green by blue, each individual pixel value *I*_*g*_(*x, y, t*) by the corresponding other pixel value *I*_*b*_(*x, y, t*)], and normalizing the videos by their minimal and maximal values, yielding motion-stabilized ratiometric videos.

### 2.6. Restitution Protocol and Arrhythmia Induction

A monophasic needle electrode (FHC Inc., USA) was inserted slightly into the epicardium of the right ventricle, firstly, to be able to apply pacing stimuli, and, secondly, to hold the heart roughly in place (with the possibility to move given the flexibility of the electrode). Restitution curves were measured using a dynamic rapid pacing protocol (Goldhaber et al., 2005) with pacing frequencies starting at *f*_*p*_ = 2.0Hz (*CL* = 500ms) and then gradually increasing until *f*_*p*_ = 10.0Hz (*CL* = 100ms). The increments were 50ms for *CL* = 500 − 350ms, then 25ms for *CL* = 350 − 260ms and 5ms for *CL* <260ms. At each cycle length, 50 pacing stimuli were applied and the last 30 elicited action potentials were analyzed. Each restitution curve measurement lasted <7 min. Typically, at the end of the pacing the hearts fibrillated. VF was terminated using an electrical defibrillation shock. A single, long optical mapping video was recorded throughout the restitution measurement. The recording was later split into individual recordings, each showing 50 action potential waves at the individual cycle lengths.

### 2.7. Post-processing

After motion tracking, warping and ratiometric combination of the green and blue videos, the resulting optical mapping videos were processed and analyzed as conventional optical mapping videos. To amplify the optical signals that correspond to action potentials, the videos were normalized using a pixel-wise, sliding-window normalization. To measure action potential durations (APDs) during pacing, time-series were extracted across the heart surface (usually averaged from 3 × 3 pixels), and the durations of 30 consecutive action potentials were measured by detecting the up- and down-strokes of each action potential at 50% or 30% height of the action potential for deriving APD_50_ and APD_70_ values, respectively. The APD was then averaged from the 30 individual APD values. To detect alternans, the durations of even and odd action potentials were measured separately from 2 × 15 action potentials. The error bars in Figure 6F show the uncertainty (standard deviation of 30 consecutive APDs in single pixel) of these measurements.

## 3. Results

**Figure 4:**
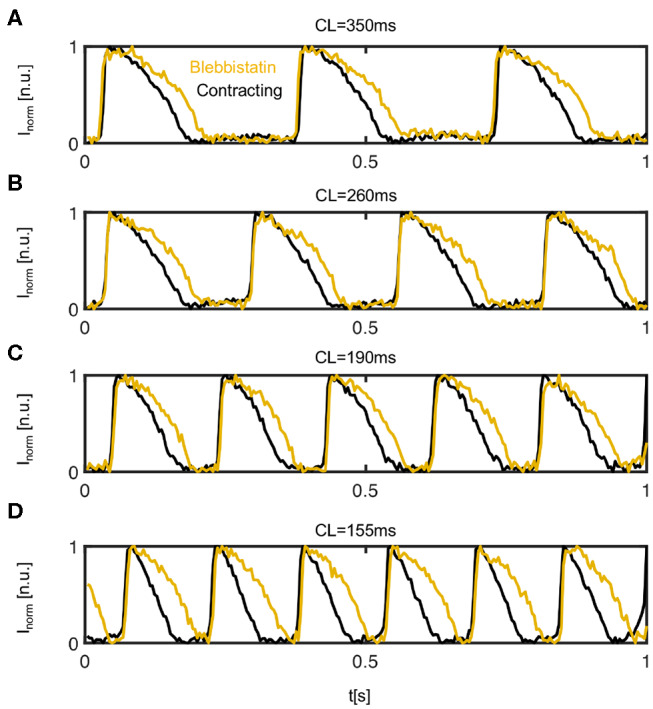
Comparison of action potentials measured optically on the left ventricular epicardium of contracting (black) vs. contraction-inhibited (yellow) isolated rabbit heart uncoupled with Blebbistatin. Representative time series obtained from 3 × 3 pixels measured in the same location on the surface of the same heart during pacing at a cycle length of **(A)** *CL* = 350*ms* (or pacing frequency *f*_*p*_ = 2.86 Hz), **(B)** *CL* = 260 ms, **(C)** *CL* = 190 ms, and **(D)** *CL* = 155 ms. The time-series were inverted and normalized (*I* ∈ [0 1]) using a sliding-window normalization.

The combination of numerical motion tracking and ratiometric imaging significantly reduced motion artifacts allowing high-resolution contact-free optical measurements of action potentials on the strongly deforming heart surface (see Figures 1C,E, 3, 4). While we found significant differences in the ventricular electrophysiology comparing contracting with Blebbistatin-arrested hearts (*N* = 10 hearts), see following sections, during sinus rhythm we found both de- and increases in the heart rate.

### 3.1. Action Potential Shortening in Contracting Hearts

Figure 4 shows four representative time-series of action potentials (AP) measured optically on the contracting and non-contracting left ventricular epicardium before (black) and after (yellow) the administration of Blebbistatin at pacing cycle lengths of 350, 260, 190, and 155*ms*, respectively. For all pacing cycle lengths, APs exhibit a different morphology and are significantly shorter on the contracting than on the non-contracting heart surface. On the contracting heart surface, the AP morphology becomes increasingly triangular-shaped with increasing pacing rates (see Figure 4D). While APD shortens with decreasing pacing cycle lengths, the relative shortening between non-contracting and contracting hearts becomes more pronounced at shorter pacing cycle lengths.

### 3.2. APD-Restitution in Contracting Hearts

**Figure 5:**
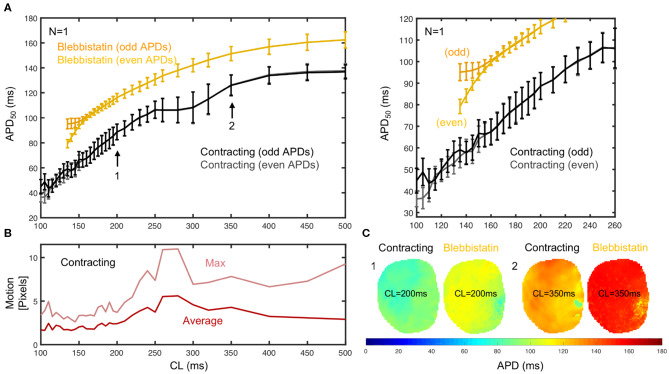
APD_50_ restitution in contracting vs. contraction-inhibited isolated rabbit heart (*N* = 1, representative example) uncoupled with Blebbistatin (at 2.8μ*M* concentration), optical measurement on left ventricular surface. **(A)** APD_50_ restitution before (black/gray) and after (yellow/orange) administration of Blebbistatin with APD_50_ measured from 2 × 15 = 30 (even and odd) consecutive action potentials at each cycle length CL (black/gray or yellow/orange = measured separately to detect alternans). Error bars indicate standard deviation of all APD_50_ in APD-maps **(C)**, or all APD_50_ measured in each pixel on heart surface. Right: Close-up of region with cardiac alternans (*CL* ≤ 160*ms*). With Blebbistatin, pacing with *CL* ≤ 140*ms* induced (non-sustained) VF. **(B)** Motion decreases with increasing pacing frequencies. Strong motion (10–20% of heart size) at *CL* = 250 − 350*ms*. **(C)** APD_50_ maps from the same heart with and without Blebbistatin for cycle lengths *CL* = 200*ms* and *CL* = 350*ms* (different heart than in **A**).

To further characterize the frequency-dependence of the APD, we measured and compared APD restitution curves of contracting vs. non-contracting hearts uncoupled with Blebbistatin (see Figure 5). A representative APD restitution measurement is shown in Figure 5A. The restitution curve measured on the contracting heart surface (black/gray) is consistently lower by about 20 − 30ms than the one obtained with Blebbistatin (yellow/orange). The relative shortening of the APD is larger at higher frequencies (~ 27% at 5Hz vs. ~ 13% at 2Hz). The measurement error is larger when hearts contract. Furthermore, in contracting hearts it was possible to measure the restitution curve up to cycle lengths of *CL* = 100ms, whereas with Blebbistatin the restitution curve exhibits cardiac alternans at cycle lengths shorter than *CL* <160ms. At cycle lengths shorter *CL* <140ms the pacing induced ventricular fibrillation (VF). Figure 5C shows exemplary APD-maps that were generated for each cycle length from the restitution measurement data.

Generally, we observed in all hearts that the overall amount of motion decreases with faster pacing rates. We also observed very strong contractions and mechanical resonance phenomena (see Figure 5B) for pacing frequencies ranging in between 3 and 4 Hz (or *CL* = 250 − 350ms). In this mid-frequency range, translational and rotational motion became large, likely due to the particular attachment and preparation of the hearts in the bath. A brief discussion of the measurement accuracy in this regime is provided in section 3.7.

**Figure 6:**
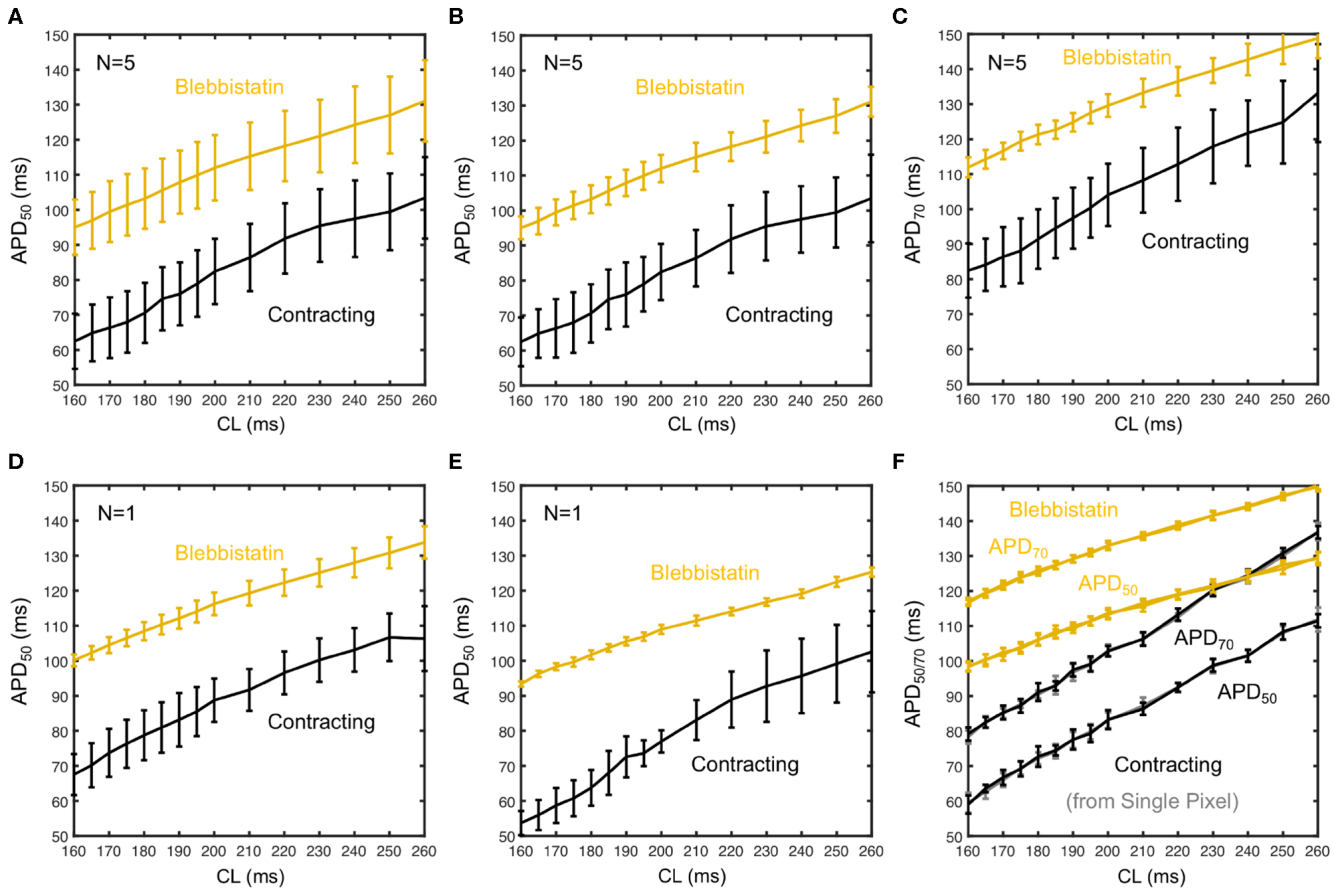
Measurement uncertainty during optical cardiac restitution measurements on left ventricular surface of contracting (black) and contraction-inhibited hearts (Blebbistatin, yellow) for *CL* = 160 − 260 ms. **(A)** Average restitution curves including variability across hearts (means and standard deviations calculated from pooled APD_50_ of *N* = 5 hearts). **(B)** Standard deviation calculated individually for each heart, mean from pooled APD_50_ as in **(A)**. Error bars indicate higher APD variability and potentially APD dispersion on the contracting heart surface. **(C)** Average APD_70_ restitution curve (*N* = 5 hearts, derived as in **B**). **(D,E)** APD_50_ restitution curves from two individual hearts, respectively. **(F)** Restitution curve measured in a single pixel (central region of field of view) from 30 consecutive action potentials (error bars: standard deviation of the 30 APDs).

Figure 6 shows averaged restitution curves, as well as restitution curves obtained from *APD*_50_ and *APD*_70_ measurements, and from single hearts and single pixels for comparison, all for both pre- (black) and post-Blebbistatin (yellow). We observed a large variability across hearts in their individual restitution characteristics. Figure 6A shows the mean restitution curves averaged from *N* = 5 hearts. The mean and uncertainty of each data point was computed as the average and standard deviation of APDs across all pixels from all 5 hearts (pooled), respectively. Correspondingly, the error bars are large, as they include the variability across different hearts. Figures 6D,E show two exemplary individual restitution curves from two different hearts, indicating a Δ*APD* ~ 10ms between hearts. Figures 6B,C show the average restitution curves derived from *N* = 5 hearts based on APD_50_ and APD_70_, respectively. The curves in Figures 6B,C show that APD_70_ provides APD values that are on average more than Δ*APD* > 10ms longer than measuring APD_50_. Correspondingly, the actual APD, which could be measured for instance at APD_90_, might be about 20ms longer than the APD_50_ values presented mostly throughout this study. Due to noise and residual motion artifacts, we did not measure APD_90_ (see also section 3.7). In contrast to Figure 6A, the error bars in Figures 6B,C were computed individually per heart (standard deviation of individual APD-maps with ~ 3, 000 - 5, 000 values) and then averaged. The error bars could both suggest that either the measurement on the contracting heart surface is less precise than with Blebbistatin, and/or that the APD variability on the contracting heart surface is greater than with Blebbistatin. However, the APD-maps in Figure 5C do not indicate stronger APD heterogeneity or gradients on the contracting heart surface, at least not within the uncertainties of the measurement. Figure 6F shows restitution curves from a single pixel, the error bars and the smoothness of the curve demonstrating the reproducibility of the measurement within a single pixel for APD_50_ and APD_70_, respectively. In summary, we found that APDs are on average 25 ± 5% shorter on the left ventricular surface of contracting than in Blebbistatin-arrested isolated rabbit hearts.

### 3.3. Blebbistatin Concentration and Washout During Pacing

**Figure 7:**
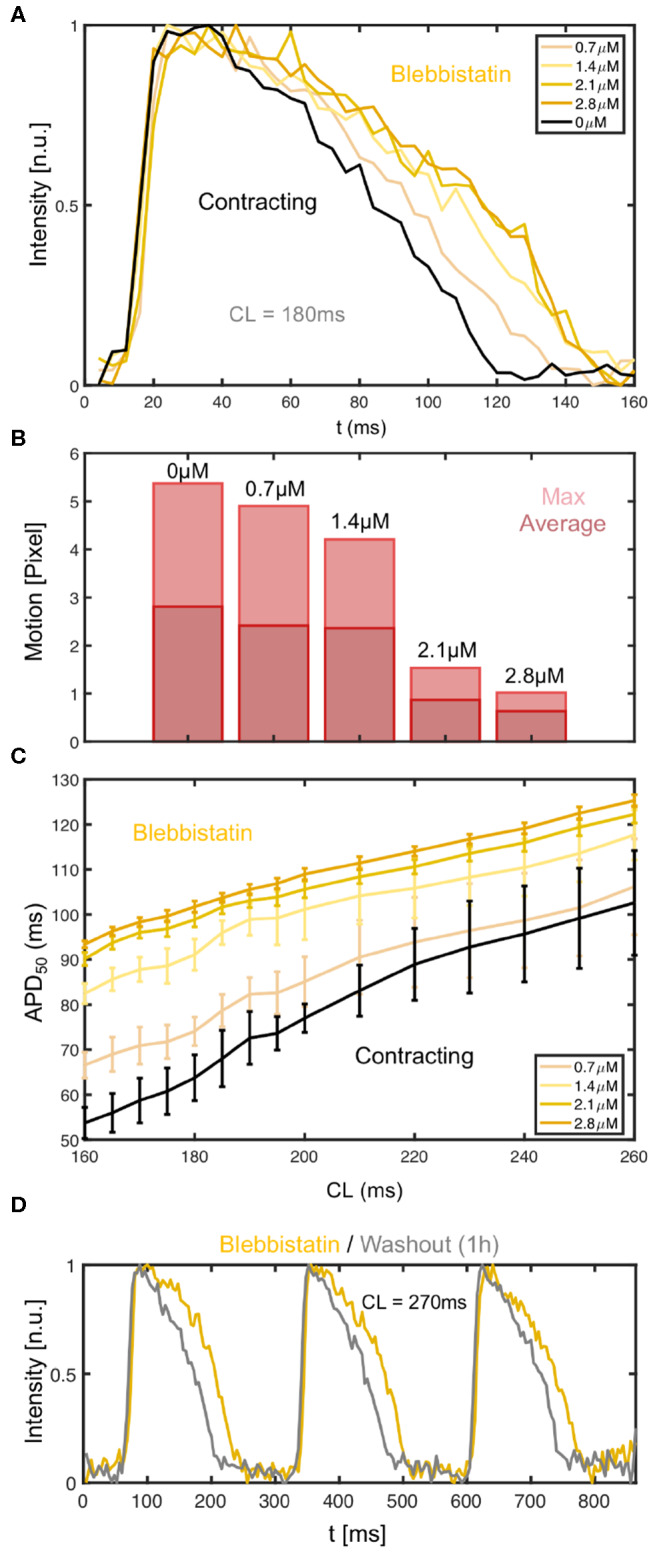
Dependence of action potential duration (APD) and restitution curve on Blebbistatin concentration. **(A)** APD prolongation with increasing Blebbistatin concentrations (black: 0μ*M* contracting, light to dark yellow: 0.7 − 2.8μ*M* Blebbistatin) at a cycle length of *CL* = 180*ms*. Optical traces obtained from 3 × 3 pixels (spatial averaging), single action potentials without temporal averaging. **(B)** Contractile motion at different Blebbistatin concentrations: baseline 0μ*M* at *CL* = 180*ms*, decreasing contractility with 0.7 − 2.8μM. **(C)** Blebbistatin concentration-dependence of restitution curves: increasing Blebbistatin concentrations prolong the APD over all cycle lengths (*N* = 1 heart, APD_50_, black: 0μM contracting, light to dark yellow: 0.7 − 2.8μM Blebbistatin). The error bars become smaller with increasing Blebbistatin concentrations and decreasing contraction magnitudes. **(D)** Shortening of action potential after washout (1h) of Blebbistatin (gray) at cycle length of *CL* = 270ms on left ventricular surface.

We found that the effect of Blebbistatin during pacing is concentration dependent, as increasing concentrations of Blebbistatin increasingly prolong the action potential (see Figures 7A–C). Figures 7A,C both show that for step-wise increases of Blebbistatin concentrations to 0.7 and 1.4μ*M* the action potential becomes subsequently prolonged, until for concentrations of 2.1 or 2.8μM the prolongation effect appears to saturate. Interestingly, the contractile motion, which is shown in Figure 7B for the different concentrations at the same cycle length as in Figure 7A, has decreased the most between 1.4 and 2.1μM, suggesting that the prolongation effect becomes smaller at higher Blebbistatin concentrations, because most of the contractile motion is already largely inhibited. Note that the Blebbistatin concentration was increased consecutively, and that the measurement might contain cumulative effects. The motion was computed by averaging over the displacements in each frame and then computing either the maximum or the average over the video sequence for each concentration.

The Blebbistatin-mediated APD prolongation is reversible since APDs shorten after washout of Blebbistatin (see Figure 7D). The action potentials were measured at a pacing frequency of *f*_*p*_ = 3.7Hz (*CL* = 270ms), at first on the surface of a Blebbistatin-arrested heart (yellow) at a concentration of 2.8μM, and subsequently in the same heart after replacing the Tyrode with Blebbistatin-free Tyrode (gray). The Tyrode was completely replaced and washed out for at least 1h with Blebbistatin-free Tyrode before the second measurement was conducted. The Tyrode containing Blebbistatin was not recirculated.

### 3.4. Electromechanical Response During Pacing

**Figure 8:**
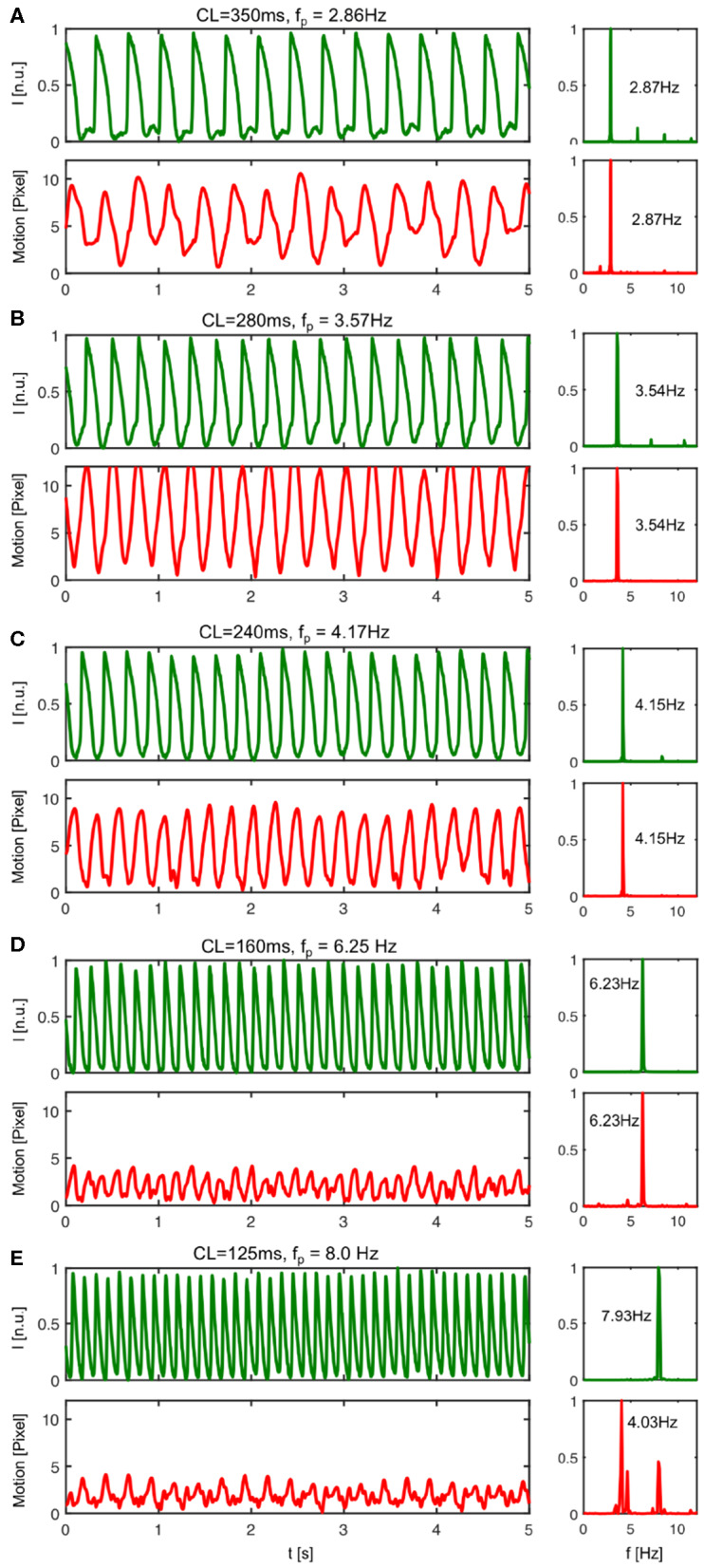
Mechanical response to electrical activation during pacing. The pacing fully captures both electrical and mechanical activation of the heart up to a frequency of about *f*_*p*_ <8Hz. In particular, for pacing frequencies below *f*_*p*_ <8Hz the mechanical contraction and motion of the heart is enslaved to the electrical pacing. Each electrical pacing stimulus leads to a contraction and equally a mechanical forth and back motion. **(A)** At pacing frequencies below *f*_*p*_ = 3Hz (here *CL* = 350ms or *f*_*p*_ = 2.86Hz) the electrical activation leads to moderately strong motion. **(B)** The motion amplitude becomes maximal for pacing frequencies ranging in between 3 − 4Hz (see also Figure 5B). At pacing frequencies above *f*_*p*_ = 4Hz (here *CL* = 240ms or *f*_*p*_ = 4.17Hz) the rapid periodic electrical activation leads to a highly correlated back and forth motion of the heart. A similar behavior can be observed until *f*_*p*_ <8Hz. The powerspectrum is mono-modal. **(D)** At a pacing frequency of *f*_*p*_ = 6.25Hz both powerspectra are mono-modal and exhibit a single dominant frequency. **(E)** With pacing frequencies >8Hz, the pacing still fully captures electrically, but the mechanical powerspectrum is no longer mono-modal.

We measured the mechanical response of the heart to the electrical activation during pacing. Figure 8 shows both the electrical activity as well as the mechanical motion of the heart measured optically in response to different pacing frequencies. The time-series were calculated by averaging the optical signals or displacements from all pixels in each video frame. The data shows in agreement with Figure 5B that the amplitude of the motion decreases with higher pacing frequencies, but that there is a resonant peak between 3 and 4 Hz where the motion becomes maximal. We observed this behavior in all hearts. We typically observed displacement magnitudes of about 10 pixels (~ 10% heart size) at lower frequencies as shown in Figures 8A,C, and larger displacements in between 10 and 15 pixels at frequencies in between 3 and 4 Hz, as shown in Figure 8C. At frequencies higher than 5Hz the motion typically decreased to below 5 pixels, as shown in Figures 8D,E.

However, more importantly, the data shows how the contractile motion of the heart stays synchronized or entrained with the electrical activation for all pacing frequencies or cycle lengths up to a frequency of about 8Hz. Electrically, there is no loss of capture. For all pacing frequencies from 2 to 10 Hz, each pacing stimulus triggers equally an action potential wave. The sequence of action potentials does not exhibit electrical alternans and fully matches with the pacing. Correspondingly, the power spectra calculated from the electrical time-series exhibit a single peak matching the pacing frequency. The power spectra calculated from the mechanical time-series also exhibit single peaks that match both the pacing frequency and the corresponding peaks in the electrical power spectra, however, only up to a frequency of about *f*_*p*_ = 8.0Hz. Beyond 8Hz every pacing stimulus leads to a corresponding synchronous electrical activation of the ventricles, but only every second pacing pulse causes a fully noticeable mechanical response. In this regime, the mechanical power spectrum exhibits two peaks, one at the pacing frequency and one at half the pacing frequency, while the electrical power spectrum exhibits only one peak still at the pacing frequency (see Figure 8E). The data suggests the existence of calcium alternans or mechanical resonance phenomena (sub-harmonics) in this regime.

### 3.5. Accelerated Vortex Wave Dynamics During Ventricular Fibrillation in Contracting Hearts

**Figure 9:**
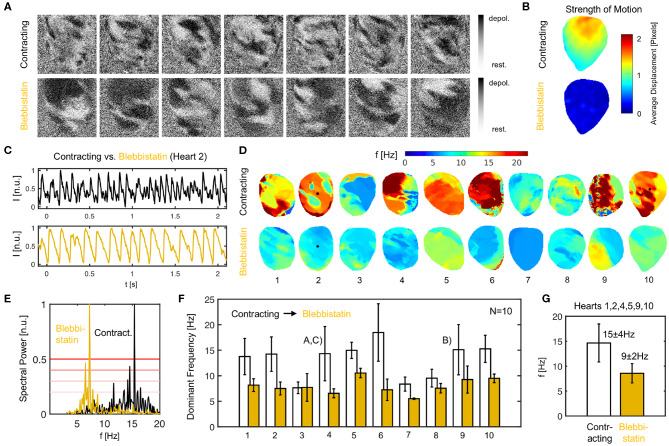
Ventricular fibrillation (VF) is faster in contracting than in Blebbistatin-arrested isolated rabbit hearts. **(A)** Raw optical mapping data (pixel-wise normalized) showing action potential waves during VF on contracting (top) and contraction-inhibited (bottom) ventricular surface, see also Supplementary Video 2. **(B)** Contractile motion without (top) and with (bottom) Blebbistatin during VF measured with numerical motion tracking (time-averaged). **(C)** Exemplary time-series (averaged from 5 × 5 pixels, inverted) showing action potential sequence on contracting heart surface (black) and with Blebbistatin (yellow). **(D)** Dominant frequency maps. **(E)** Exemplary power spectrum (cumulative from all pixels, heart 2) with and without Blebbistatin. **(F)** Frequencies in *N* = 10 hearts during 8 sec. long VF episodes without (white) and with Blebbistatin (yellow). Mean values and standard deviations computed from data in **(D,E)** including at least 5, 000 pixels containing time-series as shown in **(C)**. 4th and 9th bar plot pairs correspond to data shown in **(A–C)**, respectively. **(G)** Typical dominant frequencies during VF with and without Blebbistatin (averaged from *N* = 6 hearts).

**Figure 10:**
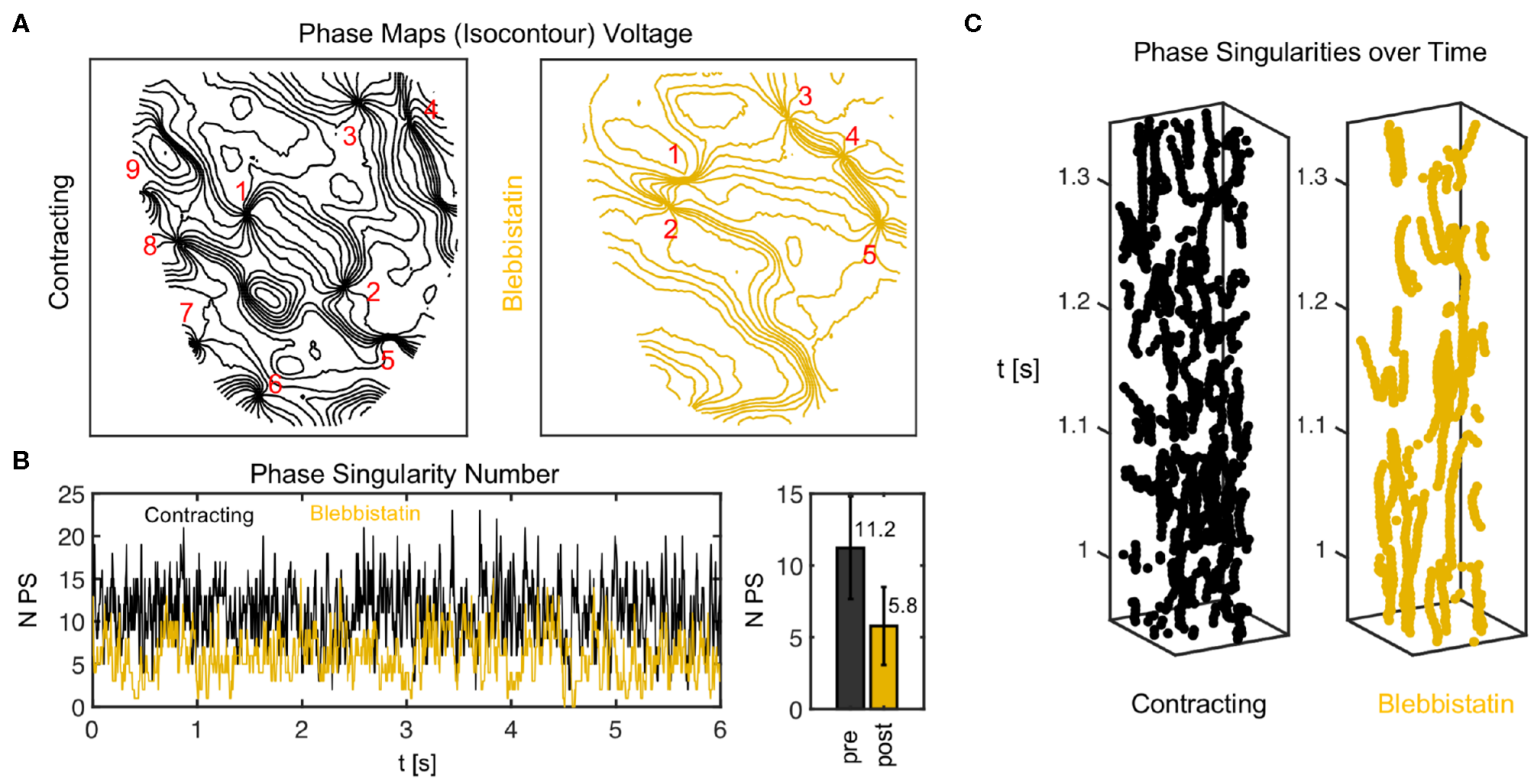
Phase singularity dynamics of action potential vortex waves on contracting and contraction-inhibited ventricular heart surface. **(A)** Phase maps showing phase singularities as nodal points. **(B)** Number of phase singularities *N*_*PS*_ over time. Right: mean of *N*_*PS*_ and standard deviation (6 s). **(C)** Space-time plots (vertical axis: time) indicating trajectories of phase singularities across heart surface.

We found that the action potential vortex wave dynamics during ventricular fibrillation (VF) become significantly altered when administering Blebbistatin. On average, contracting hearts fibrillate faster than Blebbistatin-arrested hearts (see Figure 9). Figure 9A shows two series of raw optical maps displaying and comparing the action potential wave dynamics on the surface of the same rabbit heart during VF, once contracting (top) and once Blebbistatin-arrested (bottom) see also Supplementary Video 2. To ensure comparability, the optical maps show in both cases the heart surface after numerical motion tracking and motion-stabilization, the signals being equally normalized in each pixel over time (normalized units [n.u.] ∈ [0,1], black: depolarized tissue, white: repolarized tissue). One can immediately notice that the fibrillatory waves on the contracting heart surface (top) are smaller and more fragmented than the waves on the uncoupled non-contracting heart surface (bottom), which are larger and have longer wavelengths (see also Figure 10). Correspondingly, the two exemplary optical traces or time-series in Figure 9C show shorter and more irregular action potentials on the contracting compared to the uncoupled heart surface.

We confirmed that contracting hearts typically fibrillate much faster than with Blebbistatin in *N* = 10 hearts (see Figures 9D–G). We observed frequencies ranging in the order of *f*_*c*_ = 13.1 ± 4.9Hz across the 10 hearts as they contracted, and frequencies of *f*_*B*_ = 7.9 ± 2.1Hz after they had been contraction-inhibited. The example shown in Figure 9A is typical (heart 4). However, we also observed a significant variability across hearts. While we saw a similar behavior in six hearts with frequencies decreasing from around 14.7 ± 3.8Hz down to 8.6 ± 1.9Hz with Blebbistatin (hearts 1, 2, 4, 5, 9, 10), in three cases the frequencies were already below 10*Hz* as the hearts contracted (hearts 3, 7, 8). Of these three cases, two cases (hearts 7, 8) decreased slightly further and one remained the same (heart 3). In one case, the heart did not exhibit VF but monomorphic VT with Blebbistatin (heart 7). In another case, the heart fibrillated very rapidly while contracting (~ 20*Hz*) and then the frequency fell below 10Hz with Blebbistatin (heart 6). However, in summary, in almost all cases frequencies were higher and the frequency contents were broader when hearts were contracting, as seen in Figure 9F). The frequency maps in Figure 9D show a higher spatial heterogeneity in contracting hearts and a homogenization of the activity with Blebbistatin.

It is important to point out that the VF data contains episodes that were either induced after the restitution measurements, in which the rapid pacing at the end (final pacing frequency/cycle length of 10Hz/100ms after 390 s or 6.5 min long pacing) had induced ventricular arrhythmias (hearts 1–4, 6, 9, 10), or after rapid pacing (30 − 50Hz for 30 s) with biphasic pulses (10V) during sinus rhythm (hearts 5, 7, 8). Therefore, the findings are independent from the particular pacing protocol with which the arrhythmia was induced. The perfusion pump was not turned off during the experiments.

Figure 10 shows a comparison of phase maps (isocontour lines indicating lines of equal phase) and phase singularity dynamics (nodal points) during VF without (black) and with Blebbistatin (yellow) at a concentration of 2.7μMol. The number of phase singularities is higher on the surface of the contracting than the contraction-inhibited heart, confirming the impression given by the optical maps in Figure 9A. Figure 10B shows the automatically tracked number of phase singularities over time. Overall, the number of phase singularities fluctuates strongly. However, the average number of phase singularities is higher on the contracting heart surface with *PS*_*N*_ = 11.2 ± 3.6, compared to *PS*_*N*_ = 5.8 ± 2.7 on the motion-inhibited, uncoupled surface of the same heart (over 6.0*s* or 1, 500 video frames). Note that the actual number of phase singularities on the entire surface of the heart is larger since one camera can image only 30 − 50% of the heart surface. Figure 10C shows phase singularity dynamics on the heart surface (vertical axis: time).

### 3.6. Blebbistatin Concentration and Washout During VF

**Figure 11:**
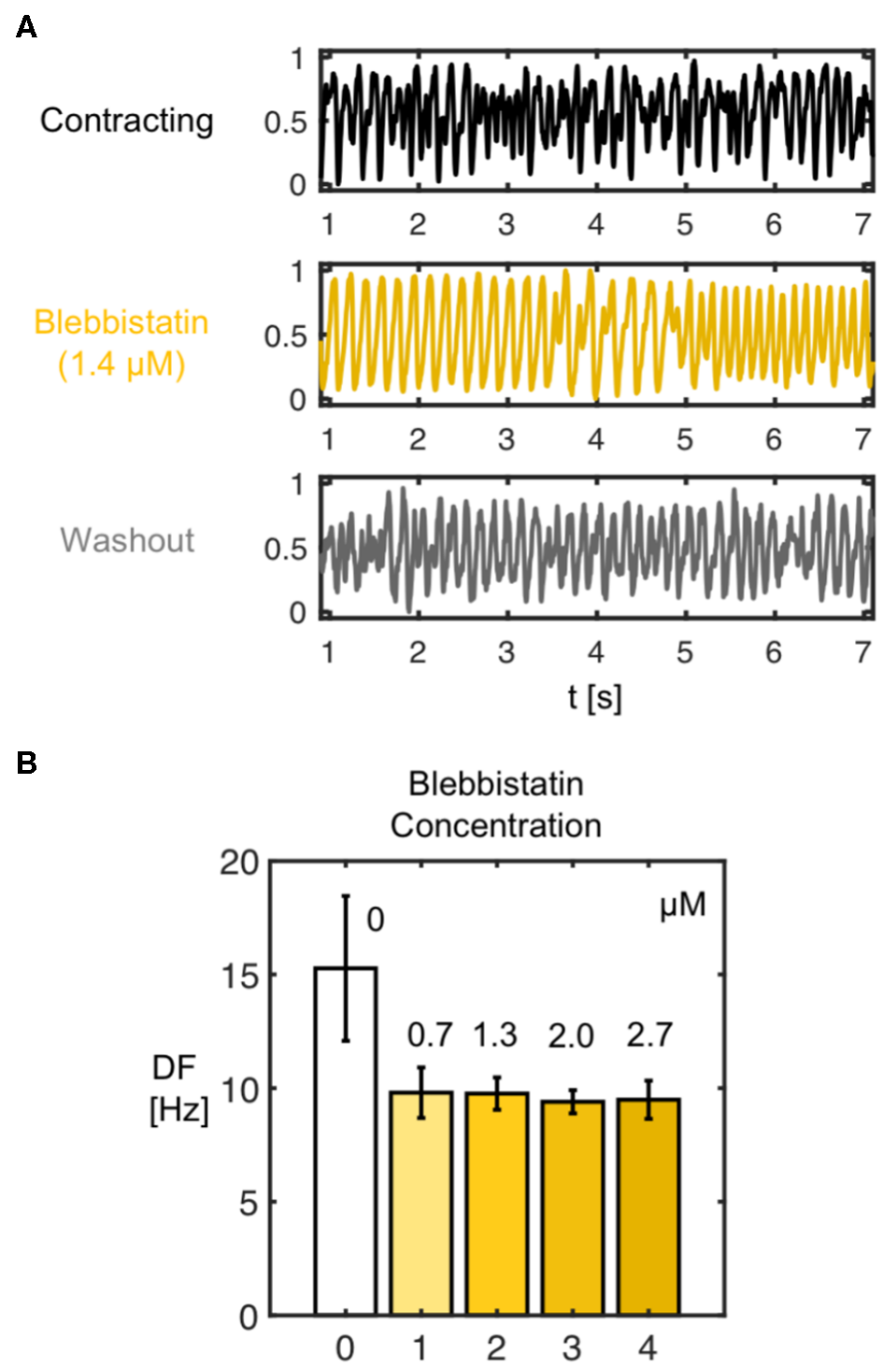
**(A)** Deceleration of VF by Blebbistatin and acceleration of VF after washout demonstrating reversibility of decelerating effect of Blebbistatin. The frequency and complexity of the electrocardiogram (shown) and optical traces measured during VF decreases with Blebbistatin (here 1.4μM after 15 min) and increases after washout of Blebbistatin (100 min), respectively. **(B)** Frequency decrease after administration of Blebbistatin (from 15.3 ± 3.2Hz to 9.6 ± 0.8Hz, data from *N* = 1 heart) to constant level for various concentrations. Frequency spectra become narrower with Blebbistatin. Confirmed for *N* = 2 hearts.

We found that the decelerating effect of Blebbistatin during VF is reversible. We performed washout experiments in *N* = 2 hearts and observed that the VF dynamics accelerate again when Blebbistatin is washed out. Figure 11A shows that when administering Blebbistatin (1.4μM concentration for 15 min) VF decelerates, and after washout with Blebbistatin-free Tyrode accelerates again (lower time-series, 100 min after starting washout). The central time-series in Figure 11A was measured 75 min after the first, and the last was measured 100 min after the central time-series. Blebbistatin was effective at suppressing contractile motion during VF at concentrations as low as 0.7μM. At 2.8μM the motion was completely suppressed (see Figure 9B). The dominant frequency during VF (computed from dominant frequency maps) decreased from about 15Hz to approximately 10Hz for all concentrations (30 min in between each measurement) (see Figure 11B).

### 3.7. Efficacy of Motion Artifact Compensation

In this study, we assumed that motion artifacts are generated mainly by two mechanisms: firstly, the dissociation or deallocation of particular tissue segments with particular pixels on the sensor, and, secondly, relative motion between tissue and light sources. Dissociation-related motion artifacts can be compensated by introducing numerical motion tracking and -stabilization, and the efficacy of the tracking was validated previously (Christoph and Luther, 2018). Illumination-related motion artifacts can be compensated using ratiometric optical mapping (Knisley et al., 2000; Bachtel et al., 2011). In this section, we discuss the advantages of the combined use of numerical motion tracking and ratiometry over numerical motion tracking and stabilization alone.

**Figure A1:**
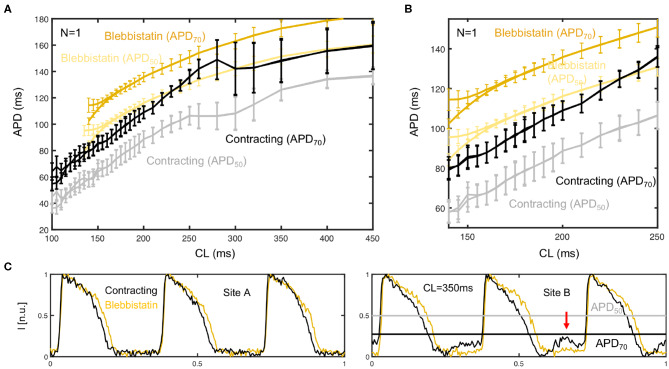
Cardiac restitution curves determined from APD_50_ and APD_70_ measurements. At 30% of the height of the action potential (APD_70_) the APD is longer than at 50% (APD_50_), but also includes larger measurement errors for lower pacing frequencies. **(A)** In this mid-frequency regime with large motion, the APDs are overestimated at APD_70_ and underestimated at APD_50_. **(B)** At higher frequencies or shorter cycle lengths *CL* <200*ms* both APD_70_ and APD_50_ values appear to provide reliable measurement data. **(C)** Corresponding time-series obtained at upper limit of mid-frequency range (*CL* = 350*ms*), which hint at measurement artifacts. In one site (site A) the optical traces look unobjectionable, whereas in another (site B) they exhibit motion artifacts that could be related to the under- or overestimation of APD shown in **(A)**.

We found that the combination of numerical motion tracking and ratiometric imaging was not always and unrestrictedly effective in compensating motion artifacts. With excessive motion even the combination of numerical motion stabilization and ratiometry faces limitations. For instance, during restitution curve measurements, we typically found a regime in which excessive motion occurred (pacing at 3 − 4Hz, displacements as large as 10 − 30% of the heart size, > 10 pixels) (see also Figure 5), and which in turn appeared to cause large measurement errors. Shows that two restitution curves that were computed from the same measurement data can strongly differ from each other and retain large uncertainties in between pacing rates of 3 − 4Hz. One curve was computed from APD_50_ and the other from APD_70_ values. Figure A1C in the Appendix illustrates how baseline modulations and deflections in the optical data may produce this under- or over-estimation (only in site B). The data suggests that either the efficacy of excitation ratiometry (different green and blue illumination fields) is limited in the presence of very strong relative (light source–tissue) motion, or other sources generating motion artifacts start to emerge with very strong contractile motion and deformation (e.g., absorption and emission changes of dye). The data demonstrates that it is yet a challenge to derive reliable restitution curves from APD_90_ values in the presence of strong motion. However, the strong translational and rotational motion that we observed in our experiments is in part caused by the specific experimental configuration, and could be avoided in future experiments (mechanical stabilization: e.g., intraventricular inflatable ballons or artificial pericard sacks).

**Figure 12:**
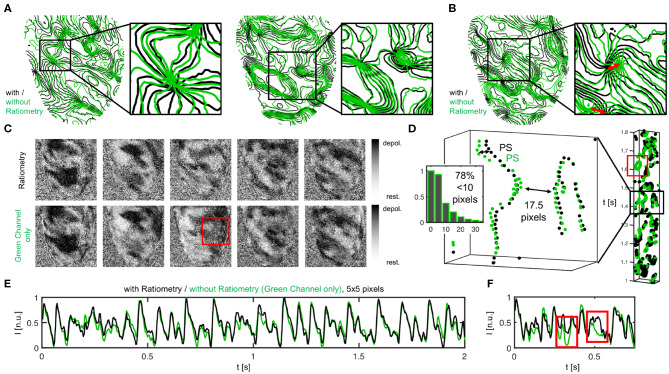
Vortex wave and phase singularity dynamics measured during VF on the contracting heart surface with numerical motion tracking and -stabilization alone (green) compared to with numerical motion tracking and -stabilization and ratiometry (black), respectively. **(A)** Good agreement between phase maps obtained with or without ratiometry, both equally showing vortex waves and vortex cores in the same locations. Phase maps are displayed as isocontour lines indicating lines of equal phase. Vortex cores or phase singular points are displayed as nodal points. **(B)** Slight mismatches and subtle differences between vortex waves and vortex core positions with and without ratiometry. **(C)** Optical maps with and without ratiometry are visually nearly indistinguishable (except for few occasions, see red box). **(D)** Space-time plot showing motion of phase singularities across heart surface over time (y-axis), once from green channel alone and with ratiometry (black). Distribution of green-black phase singularity distances (> 17, 000 phase singularities in 1, 800 video frames over duration of 7.2 s). **(E,F)** Optical traces obtained during VF with (black) and without (green) ratiometry.

During VF, subsequent ratiometric imaging does not necessarily improve the accuracy of the measurement substantially over numerical motion tracking alone, as can be seen in Figure 12. The phase maps in Figures 12A,B show fibrillatory wave dynamics measured, firstly, using numerical motion tracking and -stabilization alone (green), and secondly, using a combination of numerical motion tracking and -stabilization with ratiometry (black). Therefore, the green phase maps contain illumination-related motion-artifacts, but no or little dissociation-related motion-artifacts, whereas the black phase maps should contain neither dissociation- nor illumination-related motion-artifacts (both maps superimposed within the non-moving material coordinate frame). The high similarity between the green and the black phase maps (in 70 − 80% almost congruent and vortex cores coincide almost perfectly, see Figure 12D), in 20 − 30% subtle to moderate differences, see red arrows in Figure 12B and box in Figure 12D indicates that motion tracking is largely sufficient in suppressing motion artifacts and, vice versa, that ratiometric imaging does not necessarily improve the accuracy of the measurement much further during VF. The compensated raw optical maps (see Figure 12C), are visually nearly indistinguishable (aside from few exceptions, red box). Figure 12D shows the distribution of the mismatches (shortest distances) between the phase singularities (green-black) obtained without (green) and with (black) ratiometry (in 71% mismatches <5 pixels, in 78% mismatches <10 pixels, in 22% mismatches comparatively large > 10% pixels, average distance between both green-green or black-black phase singularities 17.5 ± 2.5 pixels). The space-time plot shows the motion of a subset of phase singularities across the heart surface (rectangular region of interest on LV to facilitate visualization) over time (y-axis, left: 1.1*s*) and illustrates that phase singularities imaged in mono-channel mode (green) represent the “true” phase singularities (black) sufficiently accurate (exception indicated by red box). The phase singularity density is almost equal (9.4 ± 4.0 without and 10.9 ± 4.0 with ratiometry in 8.0 sec. or 2, 000 video frames). The data shows that, with small amounts of contractile motion during VF, illumination artifacts become negligible and therefore tracking and numerical motion correction alone is sufficient and ratiometric imaging is not necessarily needed.

## 4. Discussion

In this study, we used marker-free numerical motion tracking and -stabilization together with ratiometric optical mapping to perform high-resolution measurements of action potential durations on the beating surface of intact Langendorff-perfused hearts, and have consequently compared the ventricular electrophysiology in beating vs. contraction-inhibited hearts uncoupled with Blebbistatin during both paced rhythms and arrhythmias. As discussed in section 3.7, we were able to retrieve largely artifact-free optical traces from high-resolution optical maps, measuring action potential waves in a co-moving frame of reference propagating across the contracting and strongly deforming heart surface. The combination of numerical motion tracking with ratiometric imaging inhibited motion artifacts substantially, enabling optical action potential duration (APD) measurements across the heart surface with uncertainties in the order of the temporal resolution of the camera. We were able to detect changes in APD in response to small changes in Blebbistatin concentrations, and we expect that we would similarly also be able to detect other physiological changes caused by, for instance, pharmacological agents. However, we observed substantial APD measurement uncertainties during excessive motion of the hearts (see Figure A1 in Appendix).

One of our main findings shows that the duration of the action potential becomes shorter in beating isolated rabbit hearts, or is prolonged in contraction-inhibited rabbit hearts uncoupled with Blebbistatin. Moreover, we found that, in agreement with the shorter APD, VF dynamics are faster and more complex in contracting rabbit hearts. Our findings confirm previous findings by Brack et al. (2013) and Lee et al. (2019), who found that ventricular electrophysiology is significantly different in beating vs. contraction-inhibited isolated rabbit and pig hearts uncoupled with Blebbistatin, respectively. More specifically, Brack et al. (2013) found that Blebbistatin substantially prolonges (~ 17 − 25%) the ventricular action potential in isolated New Zealand White rabbit hearts (*N* = 39), and increases the ventricular fibrillation threshold, or reduces the likeliness of inducing VF, at a concentration of 5μ*M*. Our study confirms the findings both directly and indirectly and we found that VF appears to be less aggressive and decelerated in uncoupled hearts, which could in principle explain higher VF thresholds. More recently, Lee et al. (2019) reported a substantial prolongation of the ventricular action potential (~ 15%), as well as a substantial decrease of the conduction velocity (~ 25%) in (*N* = 5) pig hearts *ex vivo* comparing hearts pre- and post-Blebbistatin (concentration unknown). The findings by Brack et al. (2013) and Lee et al. (2019) are in agreement with our study. Fedorov et al. (2007) reported that Blebbistatin does *not have any effect onto electrical activity*. More specifically, they found no effect onto the calcium transient morphology in single rat ventricular cardiomyocytes, and no effect onto the action potential morphology in the atria (*N* = 5) and the sinoatrial node (*N* = 5), but a slight prolongation of the action potential in the right ventricles (*N* = 6) in isolated New Zealand White rabbit hearts with Blebbistatin concentrations of 5 − 10μM at a pacing cycle length of 400*ms*. Dou et al. (2007) reported that Blebbistatin did not alter APD in single mice cardiomyocytes (*N* = 11) at a Blebbistatin concentration of 10μM. Fenton et al. (2008) found no significant change in APD in isolated equine hearts (*N* = 1) in both atria and ventricles uncoupled with Blebbistatin at concentrations of 10 − 15μM. Jou et al. (2010) reported that the action potential morphology did not get altered significantly in the atria or the ventricles of embryonic zebrafish hearts (*N* = 4) at Blebbistatin concentrations of 10μM. Lou et al. (2012) studied the electrophysiological differences between New Zealand White rabbit hearts (*N* = 7) uncoupled with BDM (10mM) vs. Blebbistatin (10μM) using panoramic optical mapping, and found that BDM produces shorter APDs than Blebbistatin. They concluded that Blebbistatin does not alter APD restitution, however, without comparing directly to a contracting heart as baseline. On the other hand, Bourgeois et al. (2011) found in isolated pig hearts (*N* = 7) *ex vivo* that BDM produces shorter APDs than when the heart contracts. Together, the findings suggest that APDs in contracting hearts are longer than with BDM, but shorter than with Blebbistatin. Lastly, Zhang et al. (2016) found at a Blebbistatin concentration of 20μM a *very similar action potential morphology* pre- and post-Blebbistatin in an isolated pig heart (*N* = 1) *ex vivo*.

A shortening of the action potential in beating isolated hearts was previously associated with a higher metabolic demand and oxygen consumption of the contracting heart muscle, which can not be sufficiently met using standard aqueous crystalloid perfusates (e.g., Tyrode) during Langendorff-perfusion (Gillis et al., 1996; Wengrowski et al., 2014; Kuzmiak-Glancy et al., 2015, 2018; Garrot et al., 2017; Ruiz and Comtois, 2018). More specifically, Gillis et al. (1996) found that APDs are shorter and ventricular fibrillation thresholds lower in crystalloid buffer-perfused vs. blood-perfused contracting rabbit hearts. Our data confirms that action potentials in beating buffer-perfused rabbit hearts become progressively triangular-shaped with faster beating rates (see Figure 4), triangularity being a hallmark of hypoxia or ischemia (Pinto and Boyden, 1999). Our data therefore strongly suggests that the beating heart's oxygen consumption becomes larger, and, consequently, an undersupply with oxygen more severe at faster beating rates. Our data is in agreement with previous findings by Garrot et al. (2017), who measured oxygen consumption to be larger and APD shorter in working than in unloaded contracting Langendorff-perfused rabbit hearts. Garrot et al. (2017) furthermore linked hypoxia and hypoxia-induced APD shortening to K_*ATP*_-channel activation, suggesting that the channel regulates contractile function in response to available oxygen and energy levels. Lastly, it was shown that crystalloid-perfused working hearts are oxygen limited and have reduced cardiac performance (Kuzmiak-Glancy et al., 2018). Other potential side-effects that seem to be associated with the use of Blebbistatin need to be considered very carefully. Brack et al. (2013) discussed potential other secondary effects of Blebbistatin that could affect APD, including vasodilatory and -constrictory phenomena that could alter perfusion and oxygen supply. Swift et al. (2012) found that Blebbistatin can form precipitate, which may block microcirculation in the cardiac muscle. In summary, the question whether Blebbistatin affects cardiac electrophysiology—directly or indirectly—remains controversial, see also Table 1, and further research is needed. As of today, it appears that one has to choose between two unphysiological conditions when performing *ex vivo* optical mapping studies in a Tyrode-based Langendorff-perfusion environment: either one can image hearts treated with Blebbistatin, which resultantly do not contract (as they are intended to), or one can renounce Blebbistatin and image beating hearts, which seem to have a higher metabolic demand than the experimental *ex vivo* environment can provide. In the future, the oxygenation problem in Langendorff experiments with contracting hearts could be addressed by using blood-based perfusion, perfusates enriched with red blood cells (or blood), or, more ideally, by using optically clear, aqueous perfusates that provide a higher oxygen carrying capacity (Kuzmiak-Glancy et al., 2018).

**Table 1:** Overview over studies investigating the effect of Blebbistatin onto cardiac electrophysiology.

**Study**	**Δ APD**	***N***	**Conc. [μM]**	**Species**	**Preparation**
Fedorov et al. (2007)	Yes	*N* = 6	5–10	Rabbit	Isolated Heart (Ventr.)
	No	*N* = 5	5–10	Rabbit	Wedge Prep. (Atria)
	No	*N* = 5	5–10	Rabbit	Wedge Prep. (SAN)
	NA	NA	5–10	Rat	Cardiomyocyte (Ventr.)
Dou et al. (2007)	NA	NA	NA	Mouse	Papillary Muscle
	No	*N* = 11	10	Mouse	Cardiomyocyte (Ventr.)
Jou et al. (2010)	No	*N* = 4	10	Zebrafish	Atria
	No	*N* = 4	10	Zebrafish	Ventricles
Fenton et al. (2008)	No	*N* = 1	10–15	Horse	Isolated Heart (Atria)
	No	*N* = 1	10–15	Horse	Isolated Heart (Ventr.)
Zhang et al. (2016)	No	*N* = 1	20	Pig	Isolated Heart (Ventr.)
Brack et al. (2013)	Yes	*N* = 39	5	Rabbit	Isolated Heart (Ventr.)
Lee et al. (2019)	Yes	*N* = 5	NA	Pig	Isolated Heart (Ventr.)
Kappadan et al.	Yes	*N* = 10	3	Rabbit	Isolated Heart (Ventr.)

*Overall, the findings regarding APD difference (Δ APD) with and without Blebbistatin remain inconclusive and seem to depend on preparations and species*.

Electrophysiological differences between beating and contraction-inhibited hearts could also be mechanically-induced and result from physiological processes unrelated to oxygenation or potential side-effects of pharmacological agents. For instance, it was observed in bypass surgery patients that APD shortens during recurring contractile work of the heart muscle when switching from “nonworking” to “working” conditions during reperfusion (Taggart et al., 1988), suggesting that mechano-electrical feedback phenomena may also influence APD. Thompson et al. (2011) found in cardiac cell cultures that using mechanosensitive channel blockers, which suppress the influence of tissue deformation on cardiac electrophysiology, similarly results in an increase of action potential conduction velocity as using Blebbistatin, suggesting that the suppression of mechanical deformation itself as well as the heart's mechano-sensitivity to it may also play a role in altering cardiac electrophysiology. Contractile work, or the absence of it, could affect various interactions between the contractile machinery, calcium handling, and excitability of cardiac cells, and strongly influence coupling and feedback mechanisms between voltage, calcium and mechanics on cellular and tissue levels.

## 5. Conclusions

We demonstrated that ratiometric electromechanical optical mapping can be used to reliably measure action potential durations on a broad range of pacing frequencies and at very high spatial and temporal resolutions on the surface of contracting isolated hearts. We found that action potential durations are shorter in contracting isolated rabbit hearts than in Blebbistatin-arrested isolated rabbit hearts perfused with standard Tyrode in Langendorff experiments. During ventricular fibrillation, the dominant frequency and the number of phase singularities are increased in contracting vs. non-contracting hearts. These observations are consistent with the assumption that contracting hearts experience mild hypoxia in the Langendorff-perfusion. Our findings may have important implications for future optical mapping studies.

## Data Availability Statement

The datasets generated for this study are available on request to the corresponding author.

## Ethics Statement

The animal study was reviewed and approved by the Lower Saxony State Office for Customer Protection and Food Safety (LAVES) and the Federation of European Laboratory Animal Science Associations (FELASA). The protocol was approved by the Lower Saxony State Office for Customer Protection and Food Safety (LAVES).

## Author Contributions

VK performed the experiments. VK and JC analyzed the data. JC wrote the initial and revised versions of the manuscript, drafted the figures, and supervised the experiments. JC, SL, and UP set the scientific agenda and supervised the research. ST contributed to the experiments and data analysis. IU and FF contributed to the experiments. All authors reviewed and approved the manuscript.

## Conflict of Interest

The authors declare that the research was conducted in the absence of any commercial or financial relationships that could be construed as a potential conflict of interest.

## A. Appendix

### A.1. Tyrode Solution

Tyrode solution was prepared with 15.0*l* distilled water, 130*mMol* or 114*g* sodium chloride (NaCl), 4*mMol* or 4.47*g* potassium chloride (KCl), 0.6*mMol* or 1.83*g* magnesium chloride hexahydrate (*MgCl*_2_(6*H*_2_*O*)), 2.2*mMol* or 3.36*g* calcium chloride (*CaCl*_2_), 1.2*mMol* or 2.16*g* sodiumdihydrogen-phosphate (*NaH*_2_*PO*_4_(*H*_2_*O*)), 24.2*mMol* or 30.45*g* sodium bicarbonate (*NaHCO*_3_), 12*mMol* or 32.4*g* glucose, resulting in a *pH*-level of 7.36 − 7.40.

### A.2. Computation of Dominant Frequencies and Dominant Frequency Maps

Dominant frequencies and dominant frequency maps, as seen in Figure 9D-G), were computed from raw optical maps as seen in Fig. Figure 9A). Prior to the computation of power spectra from time-series data, each optical trace was normalized over time by their maximal and minimal values in the entire time-series yielding normalized time-series with values ∈ [0,1], as shown in Fig. Figure 9C). Next, power spectra were computed from the time-series data independently for all pixels. The power spectra were then normalized by their maximal value or amplitude value of the dominant peak yielding a normalized power spectrum *P*(*f*) ∈ [0,1] as shown in Fig. Figure 9E). Dominant frequencies were then computed by setting a threshold *p*_*t*_ = 0.5, 0.4, 0.3, 0.2, …, clipping the power spectra's frequency contents below the threshold, and letting only frequencies with amplitudes greater the threshold contribute to an average dominant frequency:

(A1) $$\begin{array}{rcl} f_{d} & = & \frac{\sum_{i}f_{i}P_{i}}{\sum_{i}P_{i}} \end{array}$$

effectively averaging the dominant frequencies *f*_*i*_ with an amplitude of at least *P*_*i*_ ≥ 50%, 40%, … of the maximal peak from the power spectrum. We found that the different thresholds (*p*_*t*_ = 20% − 50%) equally yielded a robust computation of the dominant frequency, also accounting for multiple peaks in broader frequency spectra. All mean values and error bars shown in Fig. Figure 9 were computed with a threshold of *p*_*t*_ = 0.5.
